# Decoding the molecular cascade of embryonic-uterine modulators in pregnancy loss of PCOS mother- an “*in vivo”* study

**DOI:** 10.1186/s12958-022-01041-x

**Published:** 2022-12-07

**Authors:** Shivani Dhadhal, Laxmipriya Nampoothiri

**Affiliations:** Department of Biochemistry, Faculty of Science, The Maharaja Sayajirao University of Baroda, Vadodara, Gujarat 390002 India

**Keywords:** Polycystic ovary syndrome, Pregnancy loss, Progesterone, Letrozole, Mice

## Abstract

**Background:**

Polycystic ovary syndrome is associated with an increased rate of spontaneous abortion/early pregnancy loss and pups delivered to PCOS animals were abnormal. Currently, assisted reproductive technology has been used to help numerous infertile couples to have their babies. However, there is a low implantation rate after the transfer of embryos. Till now, it could not be concluded whether the reduced pregnancy rates observed were due to abnormal embryos or endometrial modification. Further, transgenic mouse models have been used to find out the molecular deficits behind early pregnancy complications. But, the deletion of crucial genes could lead to systemic deficiencies/embryonic lethality. Also, pregnancy is a complex process with overlapping expression patterns making it challenging to mimic their stage-specific role. Therefore, the motive of the current study was to investigate the probable molecular cascade to decipher the early pregnancy loss in the letrozole-induced PCOS mouse model.

**Methods:**

PCOS was induced in mice by oral administration of letrozole daily for 21 days. Following, the pregnancy was established and animals were sacrificed on the day 6th of pregnancy. Animals were assessed for early pregnancy loss, hormonal profile, mRNA expression of steroid receptors (*Ar, Pr, Esr1/2*), decidualization markers (*Hox10/11a*), adhesion markers (*Itgavb3, Itga4b1*), matrix metalloproteinases and their endogenous inhibitor (*Mmp2/9, Timp1/2)* and key mediators of LIF/STAT pathway (*Lif, Lifr, gp130*, *stat3*) were analyzed in the embryo implanted region of the uterus. Morphological changes in ovaries and implanted regions of the uterus were assessed.

**Results:**

Mice treated with letrozole demonstrated significant increases in testosterone levels along with a decline in progesterone levels as compared to control animals. PCOS animals also exhibited decreased fertility index and disrupted ovarian and embryo-containing uterus histopathology. Altered gene expression of the steroid receptors and reduced expression of *Hox10a*, integrins, *Mmp9, Timp1/3*, *Gp130* & *Stat3* was observed in the implanted region of the uterus of PCOS animals.

**Conclusion:**

Our results reveal that majority of the molecular markers alteration in the establishment of early pregnancy could be due to the aberrant progesterone signaling in the embryonic-uterine tissue of PCOS animals, which further translates into poor fetal outcomes as observed in the current study and in several IVF patients.

**Supplementary Information:**

The online version contains supplementary material available at 10.1186/s12958-022-01041-x.

## Introduction

Polycystic Ovary Syndrome, a term of infertility has been now recognized as one of the major infertility disorders around the world [1]. Etiopathology of this disorder is mainly linked to hyperandrogenism, hyperinsulinemia, infrequent ovulation, and the presence of numerous peripheral cysts in ovaries [2]. This multi etiological pathology is associated with clinical pregnancy complications, an increased rate of spontaneous abortion/early pregnancy loss, and preterm delivery [3, 4]. However, molecular alterations in PCOS pregnancy that originates from the mother, embryo, or both are still in debate.

To achieve a successful pregnancy, the first step is embryo implantation, wherein two-way communication between a competent blastocyst and receptive uterus gives rise to attachment and invasion of the embryo to the uterine epithelium, following the decidualization of the uterine stroma [5]. Each step of the initial pregnancy involves an interplay of the various signaling pathways in which synchronized production of ovarian estrogen and progesterone mediates structural and functional changes in the uterus [6]. These gonadal hormones exert their effect through their receptors, progesterone receptor (*Pgr*) and estrogen receptor (*Esr1* & *2*) respectively, and regulate cell proliferation, differentiation, and secretory protein production in the uterus [7]. In addition, androgen and its receptor (*Ar*) could modulate uterine growth, antagonize the expression of estrogen-regulated genes, and also helps in the decidualization of the uterine stroma [8]. During the adhesion phase of early pregnancy, integrins (e.g., α4, αv, β1, β3) are considered to be endometrial markers, and their expression is synchronized with the blastocyst attachment to the endometrium [9]. As a sequel to blastocyst-uterine attachment, transcription factors such as homeobox (*Hox10a* and *Hox11a*) genes are known to involve in the proliferation and differentiation of stromal cells surrounding the implanting blastocyst into a decidual cell [10]. Further, invasion starts with penetration of the embryo to the uterine wall which involves degradation of extracellular matrix (ECM) through matrix metalloproteases (MMP-2 & 9). Activities of MMPs are tightly controlled by their endogenous inhibitors, the tissue inhibitors of MMPs (TIMPs). The elaborated balance between the activation of MMPs and their inhibition by TIMPs is important for the regulation of embryo implantation [11]. Latterly, leukemia inhibitory factor (LIF), is a pleiotropic cytokine of the IL-6 family that is considered to influence ranges from embryo adhesion to the regulation of stromal cell proliferation [12]. LIF transduces its signal through the formation of a heterodimer with specific LIFR and the common co-receptor for the IL-6 family (gp130). The binding of the LIF to its receptor leads to activation of STAT3, which further has an impact on the modulation of embryo-uterine functions during embryo implantation [13].

These key modulators of the implanting embryo and uterus try to establish an appropriate milieu that is crucial for the development and survival of the fetus during pregnancy. However, ethical restrictions and a lack of mechanistic studies have excluded studies on embryo-endometrium interlinkage in PCOS patients. Transgenic mouse models have been used to understand the mechanistic roles of many key determinants in uterine biology and implantation. Even so, the role of crucial genes remains undetermined because their constitutive deletion could lead to systemic deficiencies and embryonic lethality [14]. Additionally, the implantation phase of pregnancy is complex, and overlapping expression patterns make it challenging to mimic their stage-specific roles. Hence, understanding the signaling mechanisms is central to implantation, and deciphering these pathways would help us to potentially alleviate many problems associated with infertility like PCOS. In this line, to study the early pregnancy stage, rodent models have been employed as they exhibit similar anatomical and physiological features of pregnancy as humans [15].

Our previous lab study has shown that oral administration of letrozole (0.5 mg/kg of body weight) daily for 21 days successfully induced PCOS in adult female Balb/c mice [16]. Also, the number of fetuses born to PCOS mothers was less compared to that of control animals along with defects in fetal growth and development have been observed. This suggests that there could be some alteration in the early window of pregnancy. Therefore, the present study was undertaken to investigate the probable regulatory mechanism for the organization of the embryonic-uterine network in the establishment of the early pregnancy events in a letrozole-induced PCOS mouse model.

## Materials and methods

### Reagents

Letrozole tablets-2.5 mg, marketed under the brand name letronat were procured from Natco Pharma Ltd. Ethanol was procured from HiMedia Laboratories Pvt. Ltd. All other reagents of analytical grade were purchased from Sisco Research Laboratories Pvt. Ltd., India. Hormones- testosterone, estradiol, and progesterone were assayed using ELISA kits (DBC Canada). RNAiso Plus was procured from Takara Inc. High-Capacity cDNA Reverse Transcription Kit was procured from Applied Biosystems. SYBR Green (Power SYBR Green PCR Master Mix Life Technologies, USA). Primers used in the study were designed by the primer blast tool of NCBI and synthesized by INTEGRATED DNA TECHNOLOGIES (IDT).

### Animal housing and maintenance

Thirty adult virgin (2–3 months) female Balb/c mice weighing 20-25 g were chosen for the study which was housed in a standard controlled animal care facility, in cages (four mice/cage), and maintained in a temperature-controlled room (22–25 °C, 45% humidity) on a 12: 12-hour dark-light cycle. The animals were maintained under standard nutritional and environmental conditions throughout the experiment. All the experiments were carried out between 9:00 and 16:00 hours, at ambient temperature. Experimental protocols were approved by the Institutional Animal Ethical Committee (IAEC), Department of Biochemistry, The M. S. University of Baroda, Vadodara (Ethical Approval Number - MSU/BIOCHEMISTRY/IAEC/2019/4). Also, experiments were performed in compliance with the ethical standards of the Committee for the Purpose of Control and Supervision of Experiments on Animals (CPCSEA), India.

### Drug administration and experimental design

**Fig. 1:**
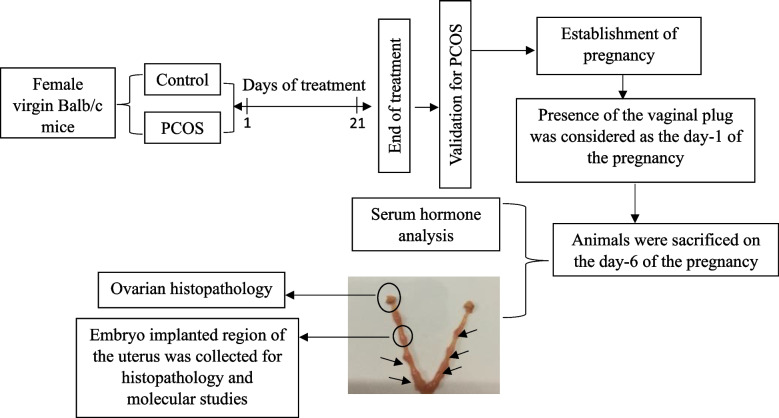
Plan of work for evaluating the pregnancy loss of letrozole-induced PCOS mouse model

Firstly, the animals were categorized into two groups-: Group I (Control group *n* = 15) received 1% Carboxymethyl cellulose (CMC) orally daily for 21 days and served as untreated Control while Group II (PCOS group *n* = 15) received letrozole (0.5 mg/kg body weight) daily for 21 days and served as PCOS. After 21 days of the treatment, PCOS validation parameters bodyweight, estrus cycle profile, hormone analysis, and ovarian histology were analyzed. Next, female mice from both groups were allowed to mate with male mice of the same strain (2:1). The following morning, females were checked for the presence of a vaginal plug. The day of vaginal plug was considered day 1 of pregnancy. On the morning of day 6 (Adhesion of embryo to uterus), animals were sacrificed. Blood samples were collected by cardiac puncture. The serum was separated and kept in a freezer at − 80 °C for determining serum hormones levels. At the end of the experiment, animals were sacrificed on the morning of day 6 of pregnancy, and the embryo containing region of the uterus was excised, trimmed, and appropriate parts were separated. One part is stored in RNAiso Plus reagent at − 20 °C for the gene expression studies and, the remaining implanted site and ovary from both the groups were dissected and stored in 10% buffered formalin, for histopathological investigations. The plan of work is provided in Fig. 1.

### Hormone estimation

Serum from blood was used as a sample to estimate the testosterone, estradiol, and progesterone levels using ELISA kits (Diagnostics Biochem Canada (DBC)- for testosterone, estradiol, and progesterone measurement). All measurements were taken according to the manufacturer’s instructions. Each sample was assayed in duplicate. The sensitivity of the kits was 0.022 ng/mL, 10 pg/mL, and 0.1 ng/mL for testosterone, estradiol, and progesterone kits respectively. The working range was 0.08 to 16.7 ng/mL, 20 to 3200 pg/mL, and 0.3 to 60 ng/mL of testosterone, estradiol, and progesterone respectively. The intra-assay coefficient of variation (CV) was 6.6 to 9.6%, 4.6 to 9.3%, and 10.2 to 10.6% for testosterone, estradiol, and progesterone kits respectively. The inter-assay coefficient of variation (CV) was 6.1 to 7.3%, 6.2 to 10.1% and 10.2 to 12.6%, for testosterone, estradiol and progesterone kits respectively. The recovery range was between 80.5 to 110.1%, 90.3 to 116.2% and 78 to 124% for testosterone, estradiol and progesterone kits respectively.

### Histological examination of the ovaries and implanted region of the uterus

The ovaries and implanted region of the uterus were fixed in 10% buffered formalin, processed, embedded in paraffin, and cut into 5-μm-thick sections. The ovarian and implanted region of the uterus sections were stained with hematoxylin and eosin and assessed microscopically. In the ovary, Graafian follicles, peripheral cystic follicles, and corpus luteum were identified. Observations and documentation were made on a DM2500 microscope (Leica, Germany) with Leica EZ digital camera under 4X magnification. In the implanted region of the uterus, attached embryo and decidual uterine cells were identified. The examination was carried out on a Nikon Ti2E microscope under 10X magnification.

### Gene expression analysis

According to previous lab protocol, relative quantification of gene expression was carried out using real-time PCR [17]. Total RNA was obtained from the implanted region of the uterus using RNAiso plus reagent as per the manufacturer’s instructions. The quantification was performed using the NanoVue Plus spectrophotometer (GE Healthcare Life Sciences) with a wavelength of 260 nm. RNA integrity was assessed by electrophoresis in a 1.2% agarose gel stained with ethidium bromide. Purity was assessed through absorption rate OD260/OD280 and samples showing a value less than 1.8 were discarded. The reverse transcription reaction to cDNA was performed using the High-Capacity cDNA Reverse Transcription Kit (Applied Biosystems) according to manufactures instructions. Real-time quantitative polymerase chain reaction (qPCR) was performed using QuantStudio 5 Real-Time PCR System using SYBR Green (Power SYBR Green PCR Master Mix – Life Technologies, USA). All samples were run in duplicate and accompanied by a non-template control. Thermal cycling conditions included initial denaturation in one cycle of 2 min at 95 °C, followed by 40 cycles of 15 s at 95 °C, 1 min at 60 °C, and 1 min at 72 °C. After amplification, the melting curves were analyzed to verify the amplification of only one product. The relative mRNA expression and fold change were calculated based on the amplification of the reference gene β-actin (ACTB). The primers used for the amplification are given in supplementary Table 1. The fold changes in expression levels of less than 0.5 and greater than 2 were considered to be biologically significant.

### Gelatin zymography

Matrix metalloproteases activity was measured as described by sohail and group [18] with modifications. All the modifications are mentioned in the procedure. A 10% tissue homogenate (Implanted region of the uterus) was prepared in PBS buffer (NaCl 0.137 M, KCL 0.0027 M, Na_2_HPO_4_ 0.01 M, KH_2_PO_4_ 0.0018 M, pH 7.1) followed by centrifugation at 10000 g for 20 minutes at 4 °C, the supernatant was collected, and the protein was estimated by lowry’s method. 80 μg of total protein samples were subjected to electrophoresis in a 10% polyacrylamide gel containing 1% gelatin. After electrophoresis, the gels were treated twice for 30 min each in 2.5% Triton X-100 and incubated for 18 h in a calcium buffer (0.05 M Tris–HCl, 0.2 M NaCl, 0.01 M CaCl_2_, 1% Triton X-100, and 1 μM ZnCl_2_, pH 7.5). Gels were stained with 0.1% Coomassie brilliant blue R-250. MMPs activities were visualized as clear bands against a dark background after distaining. The gels were then photographed and the band intensities were quantified using ImageJ software.

### Statistical analysis

The values are presented as mean ± standard error mean in all the experiments. Statistical analysis was done using student’s t-test (For Control and PCOS group) using (GraphPad Prism 5 software, La Jolla, CA). *P*-values when less than 0.05 were considered to be statistically significant at the 95% confidence limit.

## Results

### Early pregnancy loss (day-6th of pregnancy) in PCOS mice

PCOS is a complex hormonal disorder with a high risk of first-trimester miscarriage. (To validate the PCOS pathology after the 21 days of treatment, body weight, estrus cyclicity profile, hormone profile, and ovarian histology were analyzed (Supplementary Fig. 1).

**Fig. 2:**
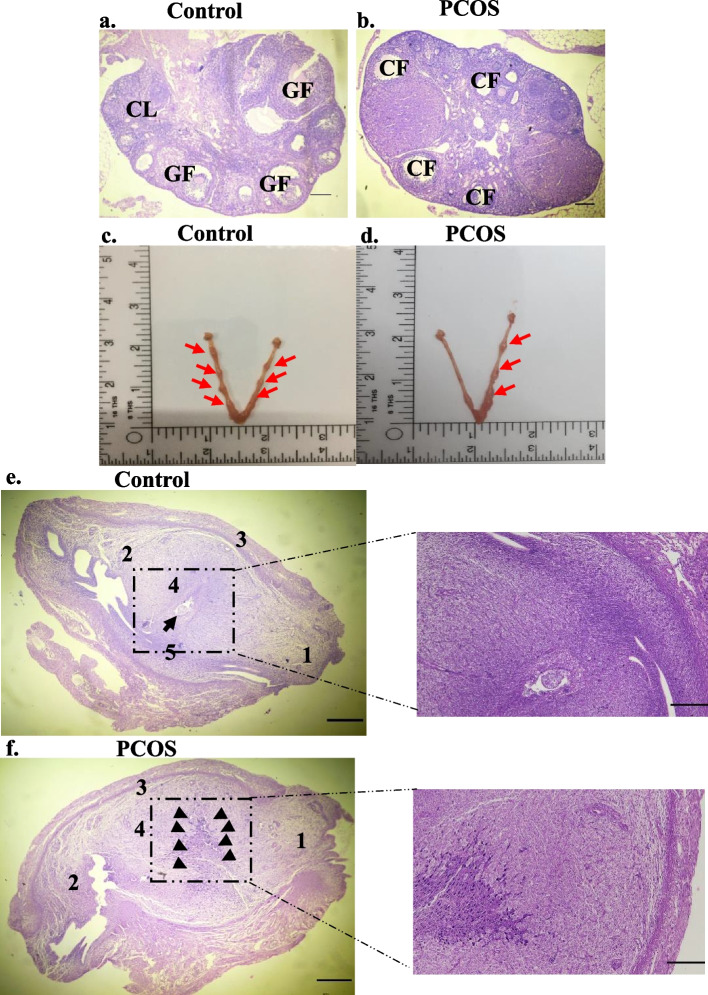
Hematoxylin and eosin-stained sections of the ovary and implanted region of the uterus. **a.** Control group **b.** PCOS group. CL: corpus luteum; CF: cystic follicle; GF- Graafian follicle, magnification 4X. Pictorial representation of a number of implanted sites. **c.** Control group **d.** PCOS group. Arrows indicate the implanting embryo. Hematoxylin and eosin-stained sections of embryo implanted region of the uterus **e.** Control group, black arrow indicates embryo **f.** PCOS group, black arrows indicate accumulation of erythrocytes. 1. Mesometrium. 2. Mesometrial endometrium. 3. Myometrium. 4. Anti-mesometrial decidua. 5. Embryo. Scale bar = 100 μm

Further, blood steroid (Testosterone, progesterone, and oestradiol) levels and ovarian histology were analyzed on day-6 of pregnancy. As shown in Table 1, the serum testosterone in the PCOS model group was significantly higher than that in the control group (**P < 0.01), which can be correlated with hyperandrogenaemia in PCOS. Also, a decrease in progesterone content was observed in letrozole-treated mice (***P < 0.001) whereas estradiol levels remained unchanged in both groups. Since it is known that maintenance of the steroid milieu is vital for the ovarian structure and function, the histology profile of the ovary using haematoxylin-Eosin stain was analyzed. The control group showed normal ovarian morphology with mature follicles (tertiary and graafian follicles) and corpora lutea. PCOS animals demonstrate multiple large peripheral cysts, fewer corpus luteum, and reduced mature follicles as compared to control animals (Fig. 2). Further, to study the implantation loss, reproductive performance, number, and histology of implanted region of the uterus were analyzed. It was observed that the number of pregnant females was reduced in letrozole induced PCOS mouse model Table 2. Also, a fewer number of implants were observed in PCOS animals compared to control animals (**p < 0.01). Figure 2 demonstrates the pictorial representation of implanted embryos in the uterus of both groups. Histology of implanted region of the uterus demonstrates the embryo has attached to the antimesometrial uterine lumen epithelium (marked by black arrows) and is surrounded by developing decidual cells in the control group. However, the PCOS group exhibited an accumulation of erythrocytes (marked by a black arrow) caused by a gain in vascular permeability was observed.

**Table 1:** Serum hormone levels on the day 6th of pregnancy

	Control	PCOS
**Testosterone** (ng/ml)	0.7020 ± 0.04903	1.240 ± 0.1435 **
**Progesterone** (ng/ml)	58.25 ± 4.029	16.00 ± 1.826 ***
**Estradiol** (pg/ml)	73.50 ± 5.377	83.00 ± 4.655 ns

The values are represented as Mean ± SEM. *N* = 6 per group. **P < 0.01, ***P < 0.001, ns-not significant as compared to Control

**Table 2:** Reproductive performances for female fertility

	Control	PCOS
**Females** (n)	14	14
**Mated females** (n)	13	12
**Pregnant females** (n)	12	8
**Not pregnant females** (n)	1	4
**Time required for conception** (in days)	3.769 ± 0.2809	3.308 ± 0.3279 ns
Mating index* (%)	92.85714 (~ 93)	85.7248 (~ 86)
Fertility index** (%)	85.71429 (~ 86)	57.14286 (~ 57)
**Total number of implants**	9.000 ± 0.3162	5.800 ± 1.020 *

The values are represented as Mean ± SEM. * *P* < 0.05, ns = not significant as compared to the control group. *Mating index = Mated females/ Total females kept for mating × 100. **Fertility index = Pregnant females/Total females kept for mating females × 100

### Altered steroid hormone receptor expression in the implanted region of the uterus in PCOS mice

**Fig. 3:**
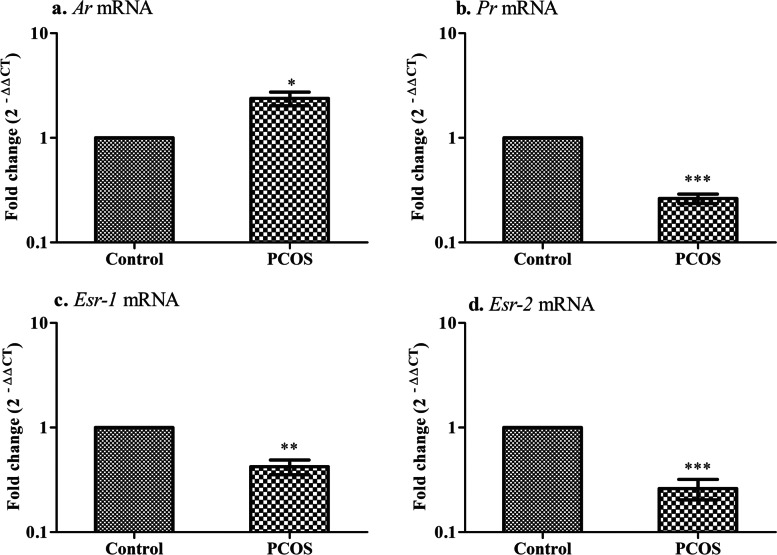
Steroid hormone receptors in the implanted region of the uterus. Values are mean fold changes in gene expression, **a.** Androgen receptor **b.** Progesterone receptor **c.** Estrogen receptor-α **d.** Estrogen receptor-β in the letrozole-induced PCOS mice model. Error bars represent SEM; *N* = 6 per group. * *P* < 0.05, **P < 0.01 ***P < 0.001 as compared to control group

To accomplish a sequential event of pregnancy, endometrium requires to undergo steroid-dependent changes. Steroids like estrogen, progesterone, and testosterone mediate their effect through their receptor’s estrogen receptor α & β (*Esr-1* & *2*), progesterone receptor (*Pgr*), and androgen receptor (*Ar*), respectively. Hence, In the implanted region of the uterus, steroid receptor transcript level was done using quantitative real-time PCR (Fig. 3). Transcriptional downregulation of *Pgr* (***P < 0.001), *Esr1* (**P < 0.01) and *Esr2* (***P < 0.001) was observed in PCOS animals. On the contrary, mRNA levels of *Ar* (* *P* < 0.05) were found markedly high in the implanted site of the uterus in PCOS animals when compared with control tissues.

### Impairment of feto-maternal interaction in the implanted region of the uterus in PCOS mice

**Fig. 4:**
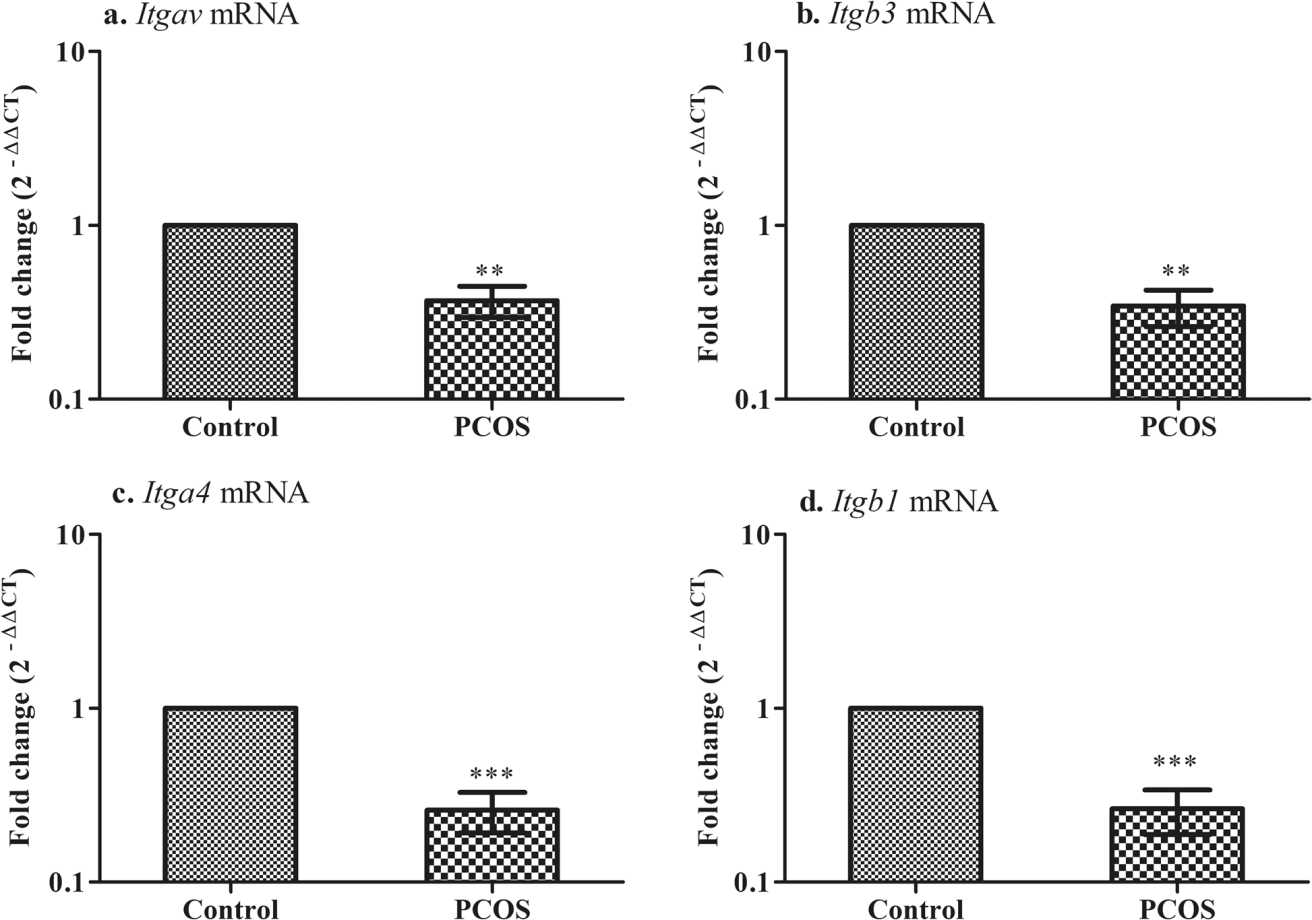
Integrin gene expression in the implanted region of the uterus. Values are mean fold changes in gene expression, **a.** Integrin- αv **b.** Integrin- β3 **c.** Integrin- α4 **d.** Integrin- β1 in the letrozole induced PCOS mice model. Error bars represent SEM; *N* = 6 per group. **P < 0.01, ***P < 0.001 as compared to the control group

Subsequently, numerous integrins - αvβ3 (*Itgav, Itgb3*), α4β1 (*Itga4, Itgb1*) are known to involve in embryo-endometrium interaction in the establishment of a healthy pregnancy. Hence, the transcript levels of these markers were evaluated in the implanted region of the uterus in the letrozole-induced PCOS mouse model. When analyzed for gene expression, *Itgav, Itgb3* (**P < 0.01), *Itga4,* and *Itgb1* (***P < 0.001) were declined in the PCOS group compared to the control group (Fig. 4).

### Modulation of decidualization events of embryo-uterine tissue in PCOS mice

**Fig. 5:**
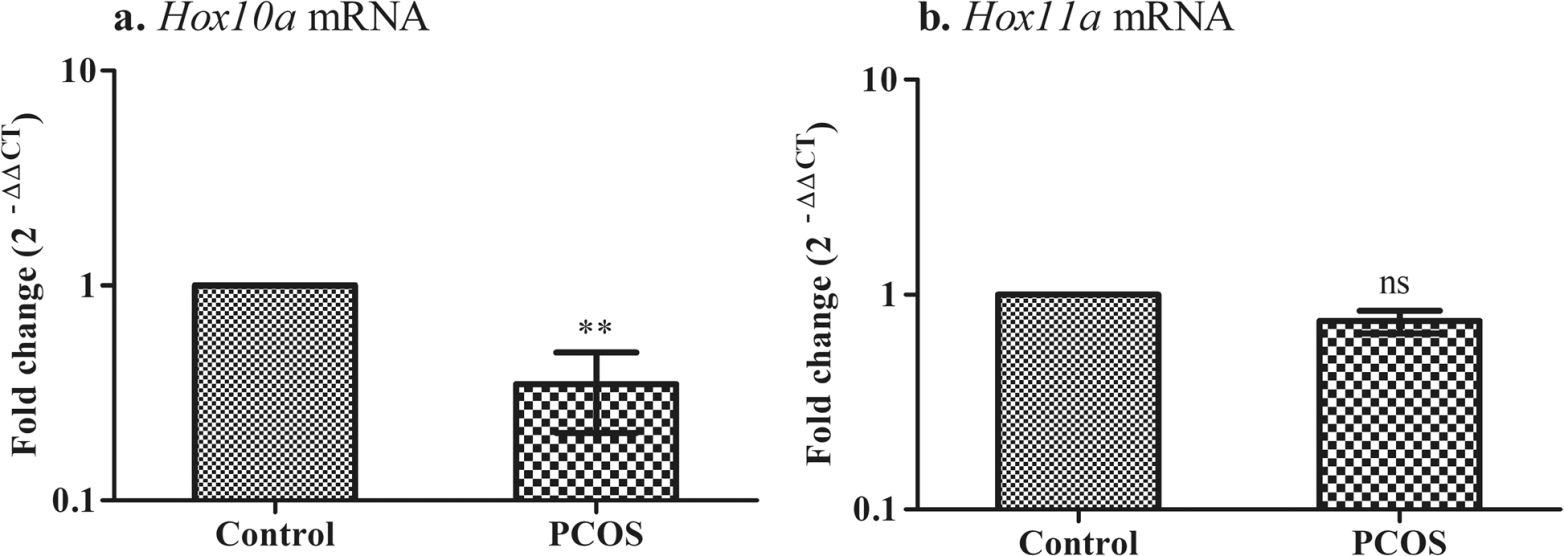
Transcription factor gene expression in the implanted region of the uterus. Values are mean fold changes in gene expression, **a.** Homeobox-10A **b.** Homeobox-11A in the letrozole induced PCOS mice model. Error bars represent SEM; *N* = 6 per group. **P < 0.01 and ns-non-significant as compared to the control group

Transcription factors, such as homeobox genes (*Hox10a*, and *Hox11a)* are known to involve in the decidualization of uterine stroma to complete the early stage of pregnancy. Hence, the transcript levels of these markers were evaluated in the implanted site of the uterus in the letrozole-induced PCOS mouse model. When analyzed for gene expression, *Hox10a* (**P < 0.01) were declined in the implanted region of the uterus in the PCOS group compared to the control group with no difference in *Hox11a* in both the groups (Fig. 5).

### Imbalance in the expression of matrix metalloproteinases and their endogenous inhibitor in the implanted region of the uterus in PCOS mice

**Fig. 6:**
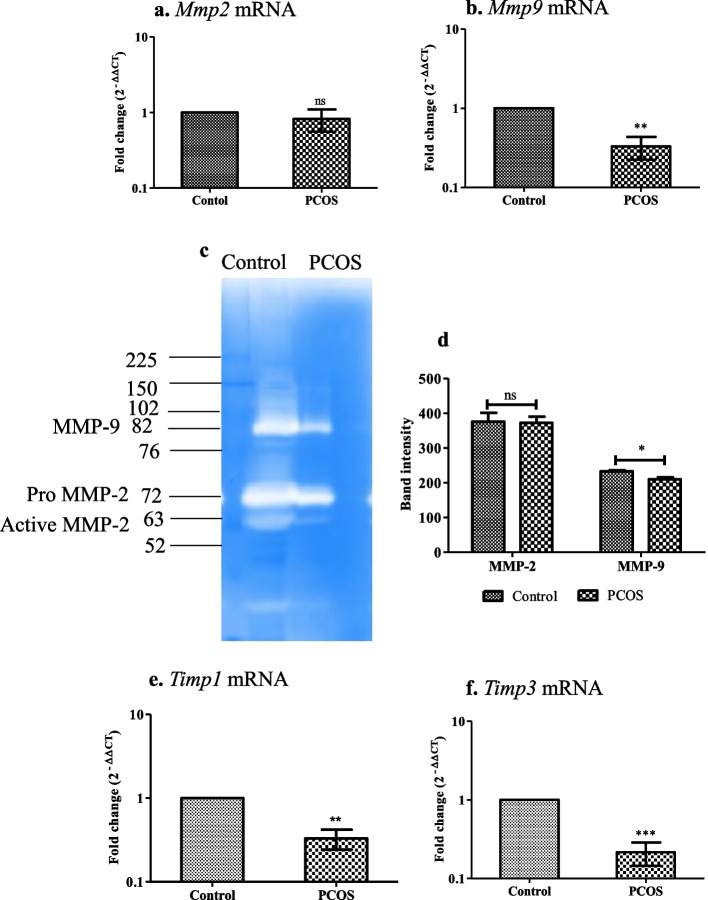
Matrix metalloproteinases and their inhibitors in the implanted region of the uterus. Values are mean fold changes in gene expression, **a.** MMP-2, and **b.** MMP-9. **c.** Gelatin gel zymograms showing pro-MMP2, active MMP2, and active MMP9 activity (arrows) in the letrozole induced PCOS mice model (represented gel picture was cropped from the same gel- uncropped/original gel picture is given in supplementary fig. S3) **d.** Quantification of total (Pro and active) MMP9 and MMP2 by computer-based densitometry analysis. **e.** & **f.** Values are mean fold change in gene expression of tissue inhibitor of metalloproteinase TIMP-1 & TIMP-3 respectively. Error bars represent mean ± SEM; *N* = 6 per group. * *P* < 0.05, **P < 0.01, ***P < 0.001, ns-non-significant as compared to the control group

Invasiveness of embryo to receptive endometrium required extensive degradation and remodeling of the extracellular matrix (ECM). Matrix metalloproteinases (*Mmp2* & *9*) are responsible for the breakdown of ECM during the implantation process. The activity of MMPs is tightly controlled by their endogenous inhibitors, the tissue inhibitors of MMPs (*Timp*). When analyzed for the transcript levels and activity of MMPs, gene expression (**P < 0.01) and activity (*P < 0.05) of MMP-9 were decreased in the implanted region of the uterus in PCOS animals compared to the control group. However, the activity of MMP-2 did not show any change in both groups. Gene expression of *Timp1* (**P < 0.01) and *Timp3* (***P < 0.001) were reduced in the implanted site of the uterus in the PCOS group compared to the control group (Fig. 6).

### Disruption of the LIF-STAT3 signaling pathway in the implanted region of the uterus in PCOS mice

**Fig. 7:**
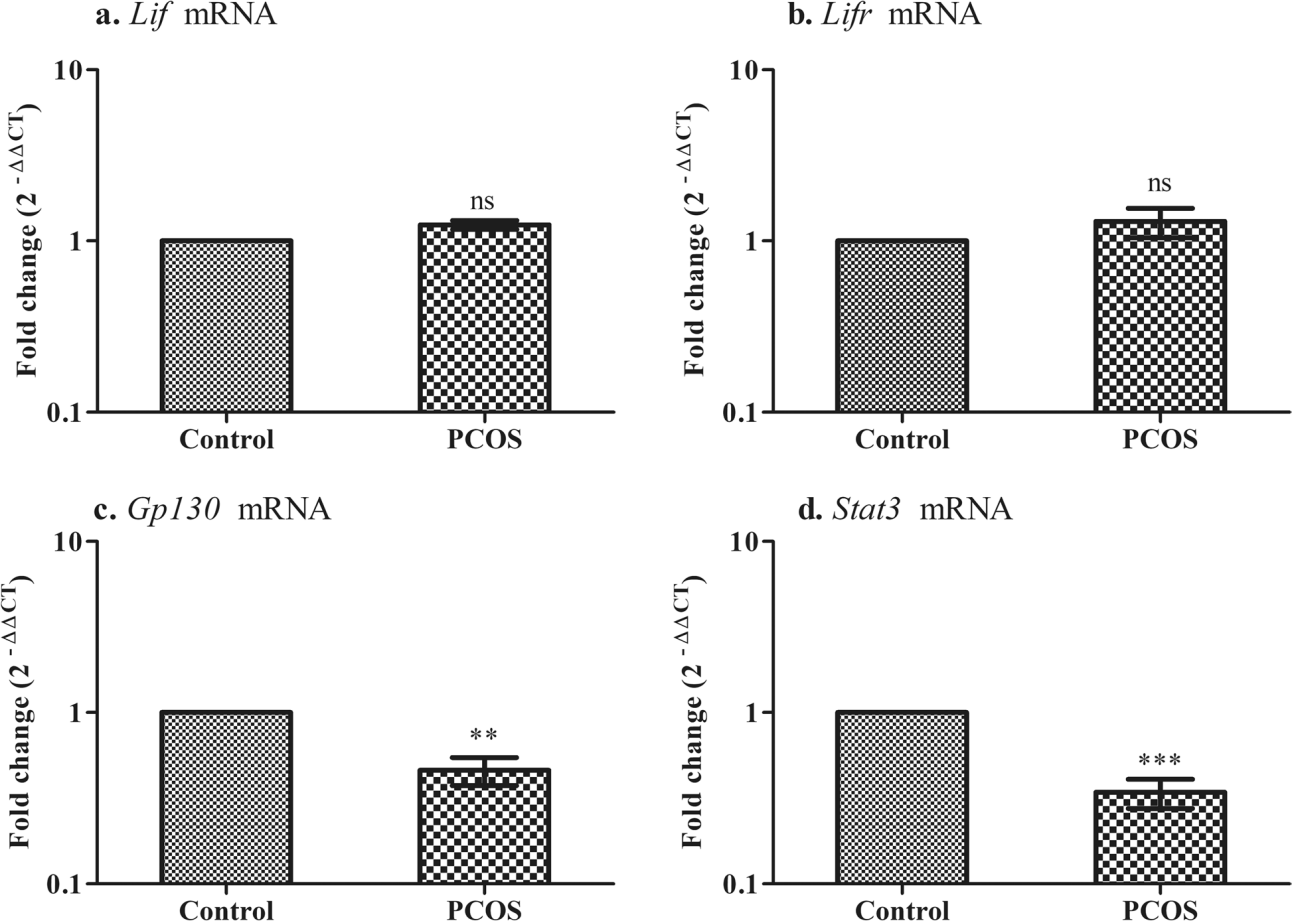
Key mediators of LIF-STAT3 related genes in the implanted region of the uterus. Values are mean fold changes in gene expression, **a.** Leukemia inhibitory factor **b.** Leukemia inhibitory factor receptor **c.** Glycoprotein 130 **d.** Signal transducer and activator of transcription 3 in the letrozole-induced PCOS mice model. Error bars represent SEM; *N* = 6 per group. **P < 0.01, ***P < 0.001, ns-non-significant as compared to control group

A tightly regulated rhythm between embryonic development and uterine maturation is essential for a successful pregnancy. This function is mediated through the cytokine on their receptors, mainly by the LIF-STAT pathway. When analyzed for key mediators of the pathway, the transcript level of glycoprotein 130 (*gp130*) (**P < 0.01) and Signal transducer and activator of transcription 3 (*Stat3*) (***P < 0.001) were declined in the implanted region of PCOS animal as compared to control group; however, no difference was observed in leukemia inhibitory factor (*Lif*) and leukemia inhibitory factor levels (*Lifr*) (Fig. 7).

## Discussion

The current study exhibited retarded fetal growth and a lesser number of pups were born to PCOS animals (Supplementary Fig. 2). In this context, early gestational events are found to be the important window that directs proper fetal growth by the numerous molecular cascade pathways. Currently, a great deal has been brought out in the domain of assisted reproductive technology (ART) and its approach could assist numerous infertile couples to have their babies. However, a major issue with this approach is the low implantation rate after several transfers of good-quality of embryos [19]. Also, it was examined that alterations in oocytes and embryos could be contributing to unsuccessful outcomes in patients with PCOS who are undergoing assisted reproduction [20]. Still, it could not be concluded whether reduced pregnancy rates seen are due to abnormal embryos which fail to implant or whether there are some modifications in the endometrium which do not allow implantation [21]. Hence, it could be noted that abnormalities in embryos and uterine independently may lead to a reduced pregnancy rate in women with PCOS. However, there are not sufficient pieces of evidence in the context of molecular alterations in the embryo containing uterine microenvironment. Thereby, the present study attempted to decode the complex mechanistic signaling of early pregnancy in a letrozole-induced PCOS mouse model.

In a previous lab study, it was reported that oral administration of letrozole (0.5 mg/kg body weight) daily for 21 days exhibits reproductive and metabolic alteration signs similar to the human PCOS condition [16]. In the current experiment, letrozole-induced female Balb/c mice showed elevated testosterone levels during early pregnancy. It could be correlated with the efficacy of letrozole (an aromatase inhibitor) in the establishment of PCOS in rodents wherein excessive accumulation of androgens occurs in the ovary [22]. In addition, low levels of progesterone were observed, which can be associated with the disruption of corpus luteum formation in PCOS animals. Similar observations were noted wherein PCOS patients are not able to form corpus luteum and generate a low level of progesterone, thus leading to abnormal ovulation [23]. Interestingly, circulating estrogen did not change in both groups, which is in accordance with studies reported in the letrozole-induced mouse model [24]. The altered hormone profile in PCOS animals might influence ovarian structure. When examined for ovarian histology, the number of peripheral cysts was observed, which is one of the characteristic features of PCOS [25, 26]. Reports have shown that hyperandrogenism and low progesterone content in women with PCOS have a lower probability of childbirth, decreased pregnancy rates, and higher miscarriage rates [27–29]. Indistinguishable, results were noticed in our study, the number of pregnant females and the number of implanting embryos in the uterus were significantly reduced in the PCOS animals. It is interesting to note that the above-cited reports have not clearly defined molecular interplay in the reduced pregnancy outcomes seen in PCOS infertility. Thereby, the present study attempts to further narrow down molecular deficits of pregnancy loss in PCOS pathology.

Progesterone signaling is known to have an inhibitory effect on the E/*Esr* signaling pathway in stromal cells of the endometrium for the establishment of pregnancy [30]. In our experimental model, the decline P/*Pgr* signaling did not show an inhibitory effect on estrogen, resulting in no difference in the estrogen-responsive gene (leukemia inhibitory factor (*Lif*)) has been observed in embryonic-uterine tissue. Furthermore, it was reported that progesterone signaling inhibited androgen receptor (*Ar)* expression, whereas estrogen dramatically elevated *Ar* abundance in the stroma of ovariectomized mouse uteri during early pregnancy [31]. In this direction, the current study revealed that altered P/*Pgr* signaling in PCOS animals did not have a prohibited influence on *Ar* expression, as the overexpression of *Ar* was observed in the implanted site of the uterus. Hence, the reduced progesterone signals in letrozole-treated animals which could be leading to dysregulated downstream targets in the implanted region of the uterus during the early pregnancy window.

Further, the study reported that when ovariectomized rats were treated with a sex-steroid regime to mimic the hormonal changes of early pregnancy, their findings have shown that progesterone is likely responsible for the regulation of αvβ3 integrin levels in the uterus [32]. Apart from steroidogenic control, LIF and its receptor are known to increase the expression of integrin αvβ3 and αvβ5 during the adhesion of the blastocyst implantation [33]. Results from the current study exhibited no difference in the gene expression of LIF & its receptor in the implanted region of the uterus in PCOS animals. This suggests that the declined expression of integrins was not mediated via LIF & LIFR. Hence, we can conclude that progesterone could be one of the contributory reasons in the reduced integrin expression, causing an impaired embryo-uterine attachment during implantation in the letrozole-induced PCOS animals.

Blastocyst attachment with the uterine epithelium is followed by the decidualization of the stromal cellsand the homeobox transcription factors are known to regulate this process [10]. Also, it was observed that progesterone and its receptor signaling upregulate the HOX10a in the isolated human endometrial stromal cells [34]. Based on this, and the above-cited references, low serum progesterone concentration in the PCOS group could not induce the HOX10a expression that is required for decidualization. As a consequence, aberrant early embryonic-uterine communication may alter the pregnancy outcomes in the PCOS phenotype.

Matrix metalloproteases (MMPs) and their inhibitors (TIMPs) have a significant role in tissue remodeling, and homeostasis of the MMPs & TIMPs is thought to be crucial during normal early gestation [35] And the importance of these proteases has been described whereas in women with an imbalance in the serum levels of MMPs and TIMPs has been associated with spontaneously terminated pregnancy in the first trimester [36]. There are reports indicating that proteases and TIMPs in the uterus have been controlled by the action of estrogen. In contrast, MMP-2 and TIMP-3 expression was not changed by steroidal treatment [11, 37]. However, in the current study, PCOS animals did not exhibit any change in estradiol content as compared to the control animals. Also, results revealed that the expression of MMP-9, TIMP-1 & 3 was significantly reduced in the letrozole-treated animals. Hence, the imbalance in the expression of the proteases and their inhibitors may be attributed to improper blastocyst invasiveness during early gestation in PCOS pathology.

Furthermore, emerging evidence suggests that ovarian steroids are reported to play a critical role in regulating the key LIF signaling markers (LIF, LIFR, and GP130) in the uterus throughout the implantation window period [12]. This is supported by the observation wherein exogenous administration of estrogen and estrogen/progesterone both can induce LIF, LIFR and GP130 expression respectively in the endometrium of ovariectomized mice [38, 39]. In addition, uterine conditional ablation of STAT3 leads to dysregulation of PR mediated pathways and decreased PR protein expression in utero*,* suggesting that STAT3 has a critical role in PR-dependent pathways during implantation in mice [40]. In this study, disrupted LIF-STAT signaling was observed in the PCOS animals. However, it couldn’t be confirmed whether the unbalanced LIF signaling could be because of altered progesterone signals or whether declined STAT3 does not activate the PR mediated pathways in the implanted region of the uterus.

Based on all of the above molecular deficits in the PCOS pregnant uterine, it was speculated that the changes observed might be originated from the modification of the histological architecture structure of the implanted region of the uterus on the day 6^th^ of pregnancy. Moreover, healthy growing implanted embryos were found in the untreated animals, whereas in the letrozole treated animals, the appearance of vascular permeability was observed in the implanted region of the uterus. These inherent changes in the structure area of the uterus could be implicated in the endometrial dysfunction in the pregnant PCOS mice.

## Conclusion

**Fig. 8:**
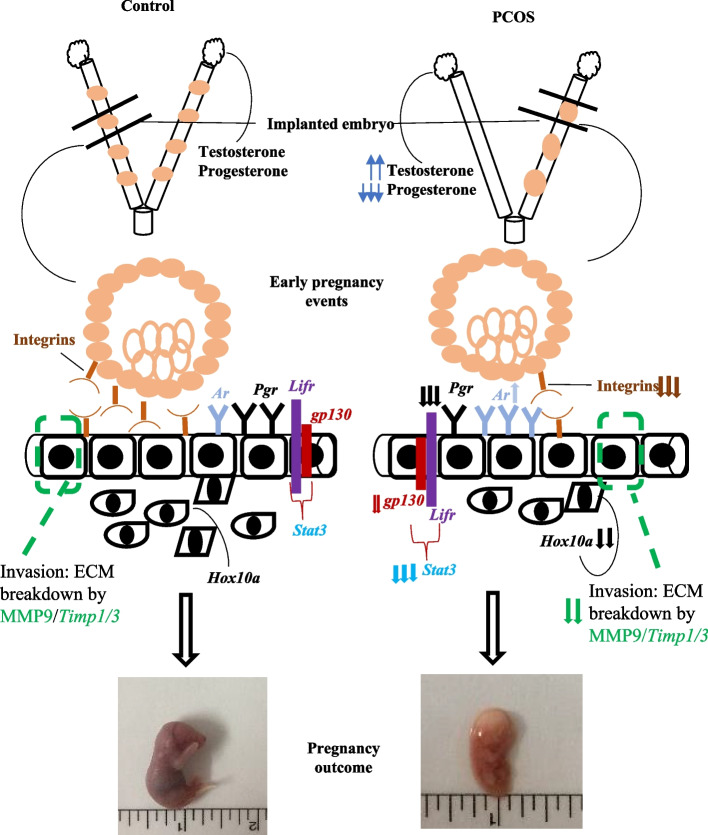
Diagrammatic summary of the current study. *Ar* androgen receptor, *Pgr* progesterone receptor, *Hox10a* homeobox transcription factor 10a, MMP Matrix metalloproteinase, *Timp* tissue inhibitor of metalloproteinase*, Lifr* leukemia inhibitory factor receptor, *Gp130* glycoprotein 130, *Stat3* signal transducer and activator of transcription 3

A significant strength of this study is to explore the potential mechanism by which PCOS may alter the embryonic-uterine microenvironment thereby preventing the establishment of a healthy pregnancy (Fig. 8). The effect of the letrozole on the implanted region of the uterus suggests that the majority of the molecular alterations were due to the aberrant PR expression and it signaling dysregulates the expression of the genes that are involved in the uterine-embryonic cross-talk during the early gestation period. Further, the abnormal expression of key markers of early gestation in PCOS could be the reason for the early pregnancy complications/early fetal loss associated with PCOS women. As evidence of the poor fertility index and reduced number of implanted embryos were observed in the PCOS animals. Thus, the current study gives insight into the regulation of intracrine molecules to improve uterine-embryonic functions and potential medicinal targets to expand the conceptive outcome of the PCOS patient.

## Supplementary Information

**Additional file 1: Supplementary Table 1.** List of primers used in the study.

**Additional file 2: Supplementary Fig. 1.** Validation of PCOS phenotype in mice.

**Additional file 3: Supplementary Fig. 2.** Defects in fetal growth and development on the day 18th of pregnancy in letrozole induced PCOS animals.

**Additional file 4: Supplementary Fig. 3.** Original/uncropped full-length gel of Fig. 6 (in the main manuscript).

## Data Availability

All data generated or analyzed during this study are included in this article [and its supplementary information files].

## Abbreviations

PCOS: Polycystic ovary syndrome
AR: Androgen receptor
PR: Progesterone receptor
ESR1/2: Estrogen receptor α/β
HOX10/11a: Homeobox 10/11a
ITGAVB3: Integrin αvβ3
ITGA4B1: Integrin α4β1
MMP2/9: Matrix metalloproteinases 2/9
TIMP: Tissue inhibitor of metalloproteinase
LIF: Leukemia inhibitory factor
LIFR: Leukemia inhibitory factor receptor
GP130: Glycoprotein 130
STAT3: Signal transducer and activator of transcription 3
