# Supplementary material for: Decoding the molecular cascade of embryonic-uterine modulators in pregnancy loss of PCOS mother- an “in vivo” study
Source: Reprod Biol Endocrinol. 2022 Dec 7;20:165. doi: 10.1186/s12958-022-01041-x (PMC9727897; doi:10.1186/s12958-022-01041-x)
Supplement: Supplementary file 1 — Additional file 1: Supplementary Table 1. List of primers used in the study. [file 12958_2022_1041_MOESM1_ESM.pptx]

## Slide 1
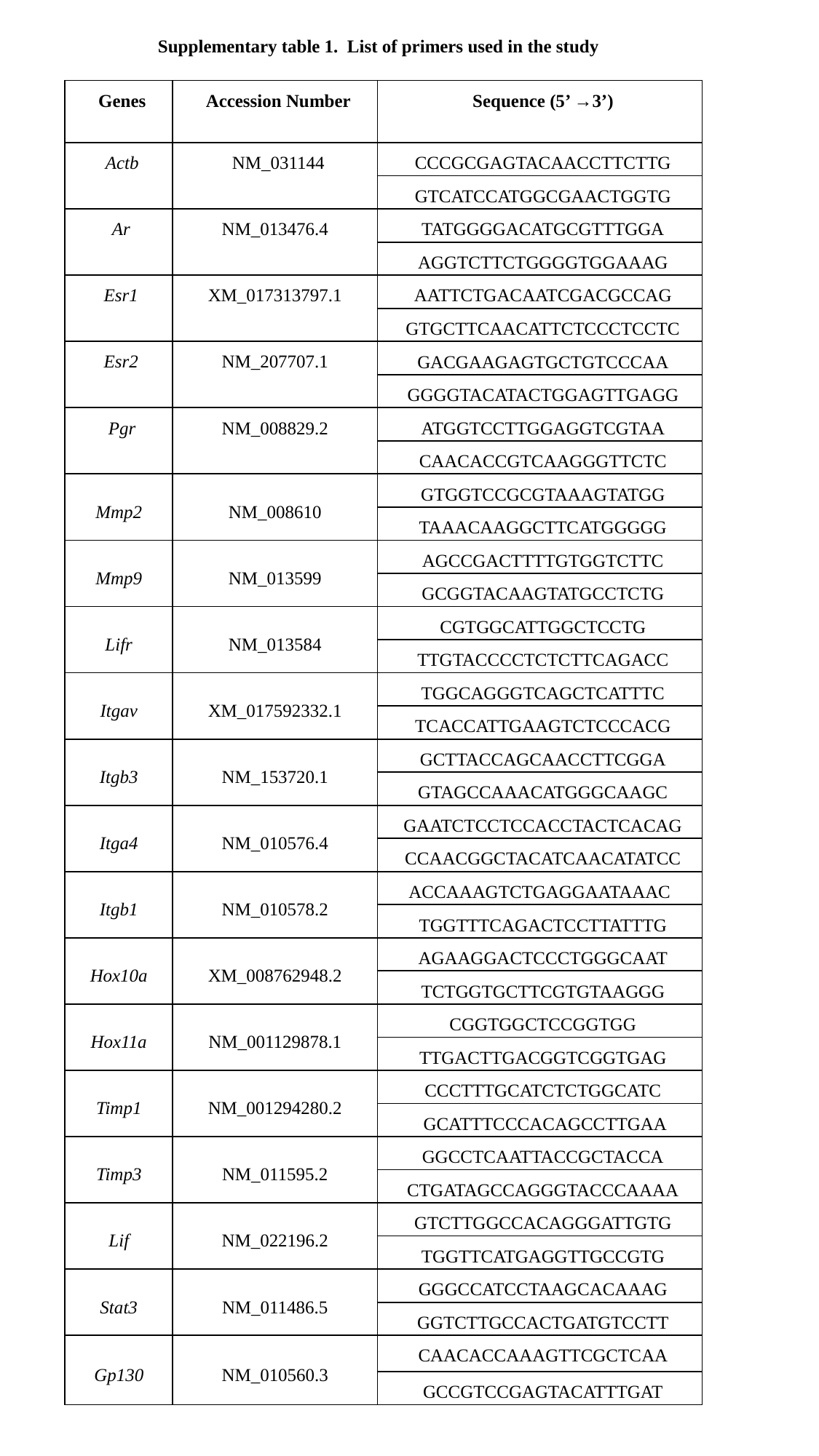

Supplementary table 1. List of primers used in the study
| Genes | Accession Number | Sequence (5’ →3’) |
| --- | --- | --- |
| Actb | NM\_031144 | CCCGCGAGTACAACCTTCTTG |
| | | GTCATCCATGGCGAACTGGTG |
| Ar | NM\_013476.4 | TATGGGGACATGCGTTTGGA |
| | | AGGTCTTCTGGGGTGGAAAG |
| Esr1 | XM\_017313797.1 | AATTCTGACAATCGACGCCAG |
| | | GTGCTTCAACATTCTCCCTCCTC |
| Esr2 | NM\_207707.1 | GACGAAGAGTGCTGTCCCAA |
| | | GGGGTACATACTGGAGTTGAGG |
| Pgr | NM\_008829.2 | ATGGTCCTTGGAGGTCGTAA |
| | | CAACACCGTCAAGGGTTCTC |
| Mmp2 | NM\_008610 | GTGGTCCGCGTAAAGTATGG |
| | | TAAACAAGGCTTCATGGGGG |
| Mmp9 | NM\_013599 | AGCCGACTTTTGTGGTCTTC |
| | | GCGGTACAAGTATGCCTCTG |
| Lifr | NM\_013584 | CGTGGCATTGGCTCCTG |
| | | TTGTACCCCTCTCTTCAGACC |
| Itgav | XM\_017592332.1 | TGGCAGGGTCAGCTCATTTC |
| | | TCACCATTGAAGTCTCCCACG |
| Itgb3 | NM\_153720.1 | GCTTACCAGCAACCTTCGGA |
| | | GTAGCCAAACATGGGCAAGC |
| Itga4 | NM\_010576.4 | GAATCTCCTCCACCTACTCACAG |
| | | CCAACGGCTACATCAACATATCC |
| Itgb1 | NM\_010578.2 | ACCAAAGTCTGAGGAATAAAC |
| | | TGGTTTCAGACTCCTTATTTG |
| Hox10a | XM\_008762948.2 | AGAAGGACTCCCTGGGCAAT |
| | | TCTGGTGCTTCGTGTAAGGG |
| Hox11a | NM\_001129878.1 | CGGTGGCTCCGGTGG |
| | | TTGACTTGACGGTCGGTGAG |
| Timp1 | NM\_001294280.2 | CCCTTTGCATCTCTGGCATC |
| | | GCATTTCCCACAGCCTTGAA |
| Timp3 | NM\_011595.2 | GGCCTCAATTACCGCTACCA |
| | | CTGATAGCCAGGGTACCCAAAA |
| Lif | NM\_022196.2 | GTCTTGGCCACAGGGATTGTG |
| | | TGGTTCATGAGGTTGCCGTG |
| Stat3 | NM\_011486.5 | GGGCCATCCTAAGCACAAAG |
| | | GGTCTTGCCACTGATGTCCTT |
| Gp130 | NM\_010560.3 | CAACACCAAAGTTCGCTCAA |
| | | GCCGTCCGAGTACATTTGAT |
