# Supplementary material for: Decoding the molecular cascade of embryonic-uterine modulators in pregnancy loss of PCOS mother- an “in vivo” study
Source: Reprod Biol Endocrinol. 2022 Dec 7;20:165. doi: 10.1186/s12958-022-01041-x (PMC9727897; doi:10.1186/s12958-022-01041-x)
Supplement: Supplementary file 2 — Additional file 2: Supplementary Fig. 1. Validation of PCOS phenotype in mice. [file 12958_2022_1041_MOESM2_ESM.pptx]

## Slide 1
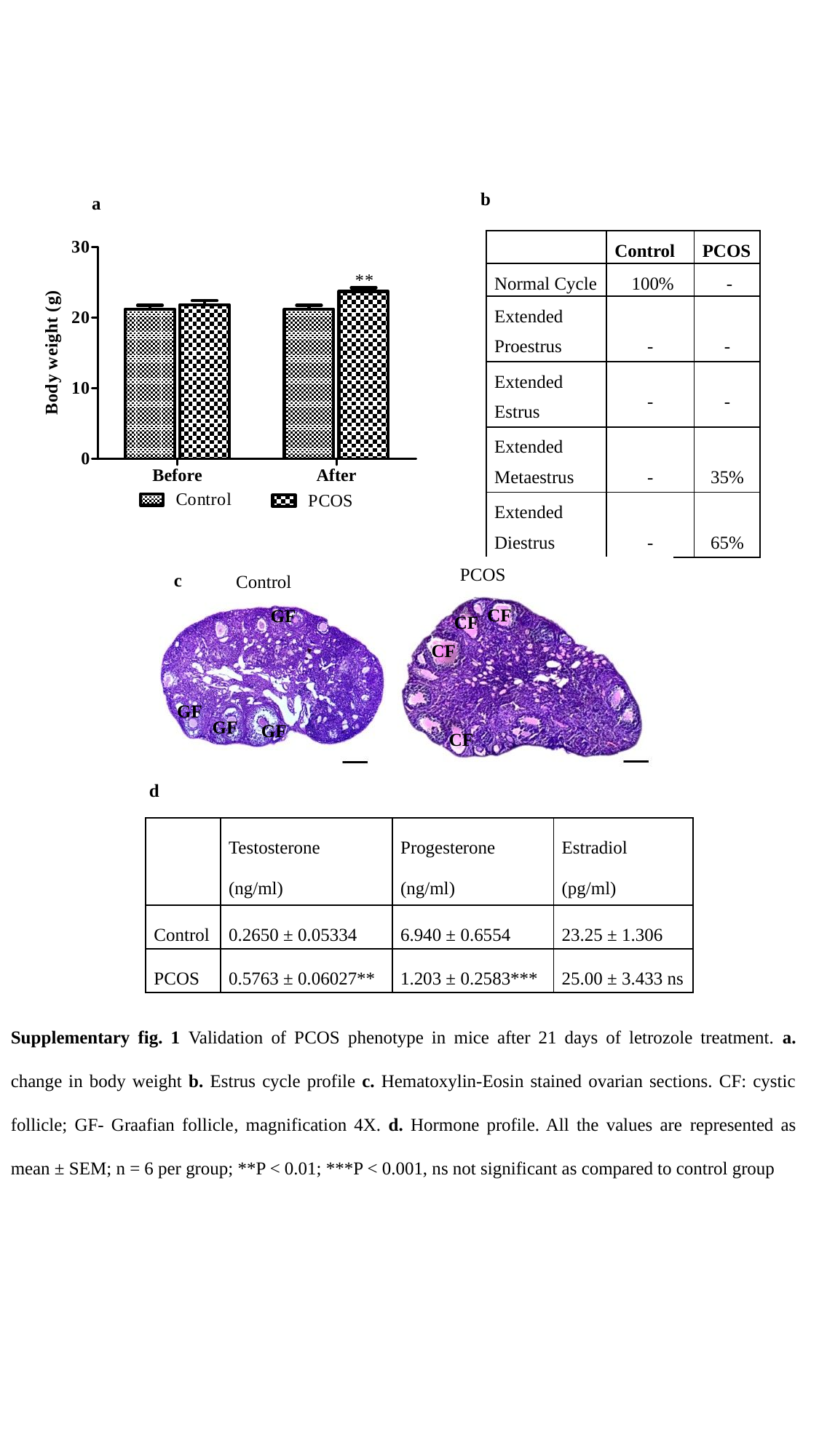

b
a
| | Control | PCOS |
| --- | --- | --- |
| Normal Cycle | 100% | - |
| Extended Proestrus | - | - |
| Extended Estrus | - | - |
| Extended Metaestrus | - | 35% |
| Extended Diestrus | - | 65% |
CF
GF
CF
CF
GF
GF
GF
CF
PCOS
c
c
Control
d
| | Testosterone (ng/ml) | Progesterone (ng/ml) | Estradiol (pg/ml) |
| --- | --- | --- | --- |
| Control | 0.2650 ± 0.05334 | 6.940 ± 0.6554 | 23.25 ± 1.306 |
| PCOS | 0.5763 ± 0.06027\*\* | 1.203 ± 0.2583\*\*\* | 25.00 ± 3.433 ns |
Supplementary fig. 1 Validation of PCOS phenotype in mice after 21 days of letrozole treatment. a. change in body weight b. Estrus cycle profile c. Hematoxylin-Eosin stained ovarian sections. CF: cystic follicle; GF- Graafian follicle, magnification 4X. d. Hormone profile. All the values are represented as mean ± SEM; n = 6 per group; **P < 0.01; ***P < 0.001, ns not significant as compared to control group
