# Supplementary material for: Decoding the molecular cascade of embryonic-uterine modulators in pregnancy loss of PCOS mother- an “in vivo” study
Source: Reprod Biol Endocrinol. 2022 Dec 7;20:165. doi: 10.1186/s12958-022-01041-x (PMC9727897; doi:10.1186/s12958-022-01041-x)
Supplement: Supplementary file 3 — Additional file 3: Supplementary Fig. 2. Defects in fetal growth and development on the day 18th of pregnancy in letrozole induced PCOS animals. [file 12958_2022_1041_MOESM3_ESM.pptx]

## Slide 1
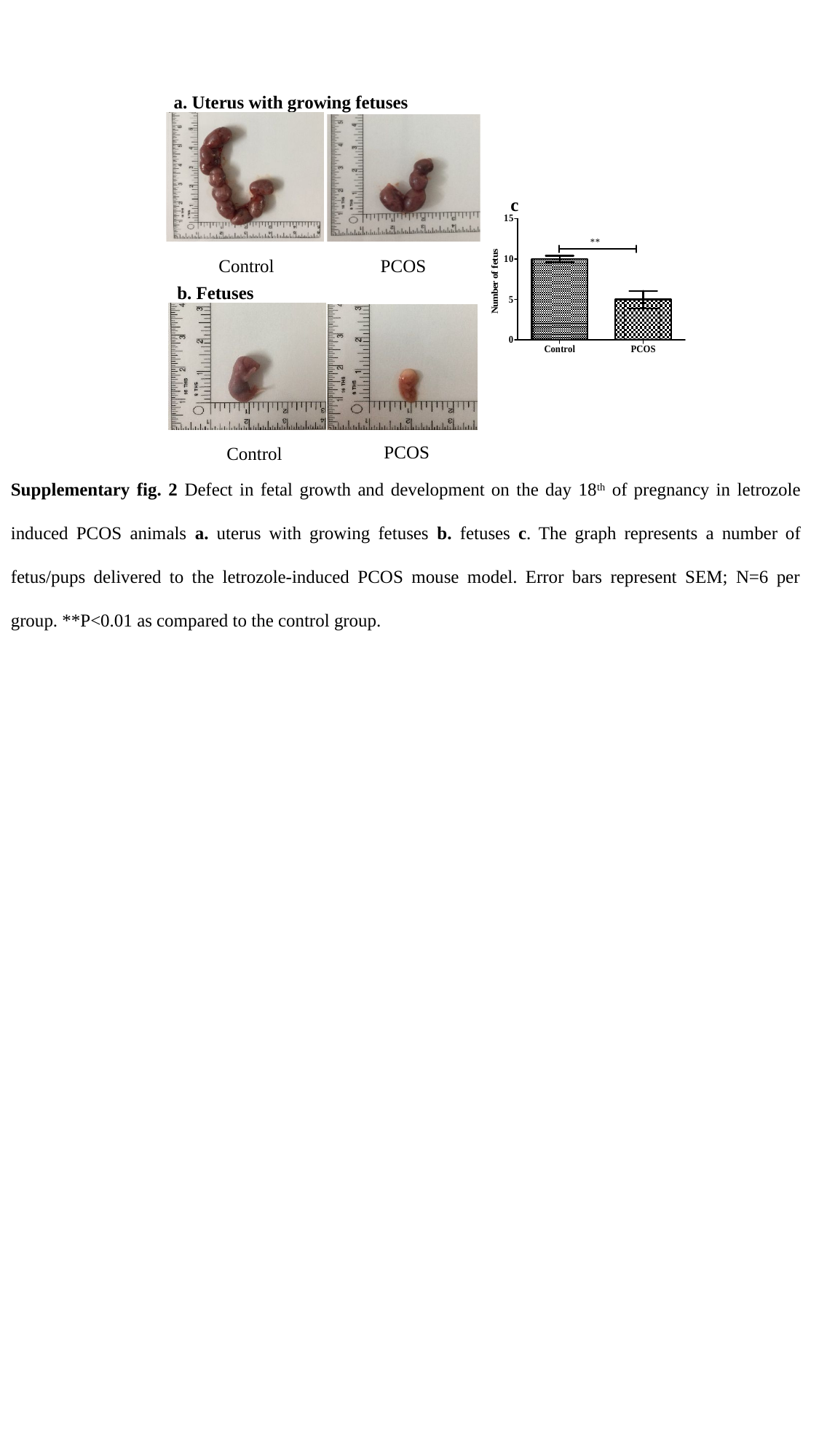

a. Uterus with growing fetuses
c
PCOS
Control
b. Fetuses
PCOS
Control
Supplementary fig. 2 Defect in fetal growth and development on the day 18th of pregnancy in letrozole induced PCOS animals a. uterus with growing fetuses b. fetuses c. The graph represents a number of fetus/pups delivered to the letrozole-induced PCOS mouse model. Error bars represent SEM; N=6 per group. **P<0.01 as compared to the control group.
