# Supplementary material for: Decoding the molecular cascade of embryonic-uterine modulators in pregnancy loss of PCOS mother- an “in vivo” study
Source: Reprod Biol Endocrinol. 2022 Dec 7;20:165. doi: 10.1186/s12958-022-01041-x (PMC9727897; doi:10.1186/s12958-022-01041-x)
Supplement: Supplementary file 4 — Additional file 4: Supplementary Fig. 3. Original/uncropped full-length gel of Fig. 6 (in the main manuscript). [file 12958_2022_1041_MOESM4_ESM.pptx]

## Slide 1
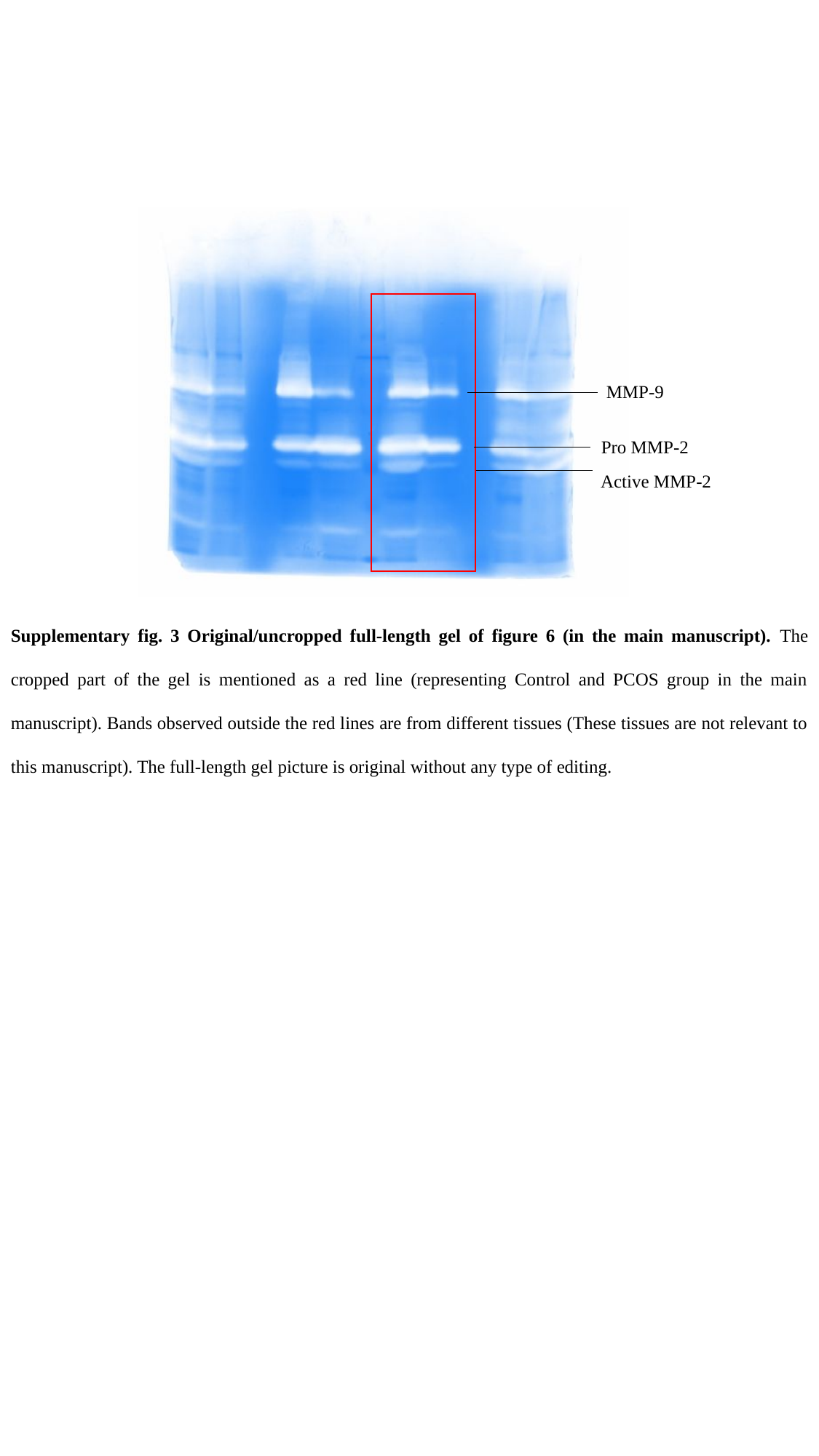

MMP-9
Pro MMP-2
Active MMP-2
Supplementary fig. 3 Original/uncropped full-length gel of figure 6 (in the main manuscript). The cropped part of the gel is mentioned as a red line (representing Control and PCOS group in the main manuscript). Bands observed outside the red lines are from different tissues (These tissues are not relevant to this manuscript). The full-length gel picture is original without any type of editing.
